# A framework for understanding post-detection deception in predator–prey interactions

**DOI:** 10.7717/peerj.15389

**Published:** 2023-06-23

**Authors:** Juliette J. Rubin, Akito Y. Kawahara

**Affiliations:** 1 McGuire Center for Lepidoptera and Biodiversity, Florida Museum of Natural History, University of Florida, Gainesville, FL, USA; 2 Department of Biology, University of Florida, Gainesville, FL, USA

**Keywords:** Sensory ecology, Object formation, Evolutionary drivers, Perception, Sensory illusions

## Abstract

Predators and prey exist in persistent conflict that often hinges on deception—the transmission of misleading or manipulative signals—as a means for survival. Deceptive traits are widespread across taxa and sensory systems, representing an evolutionarily successful and common strategy. Moreover, the highly conserved nature of the major sensory systems often extends these traits past single species predator–prey interactions toward a broader set of perceivers. As such, deceptive traits can provide a unique window into the capabilities, constraints and commonalities across divergent and phylogenetically-related perceivers. Researchers have studied deceptive traits for centuries, but a unified framework for categorizing different types of post-detection deception in predator–prey conflict still holds potential to inform future research. We suggest that deceptive traits can be distinguished by their effect on object formation processes. Perceptual objects are composed of physical attributes (what) and spatial (where) information. Deceptive traits that operate after object formation can therefore influence the perception and processing of either or both of these axes. We build upon previous work using a perceiver perspective approach to delineate deceptive traits by whether they closely match the sensory information of another object or create a discrepancy between perception and reality by exploiting the sensory shortcuts and perceptual biases of their perceiver. We then further divide this second category, sensory illusions, into traits that distort object characteristics along either the what or where axes, and those that create the perception of whole novel objects, integrating the what/where axes. Using predator–prey examples, we detail each step in this framework and propose future avenues for research. We suggest that this framework will help organize the many forms of deceptive traits and help generate predictions about selective forces that have driven animal form and behavior across evolutionary time.

## Introduction

### Introducing the framework

**Figure 1:**
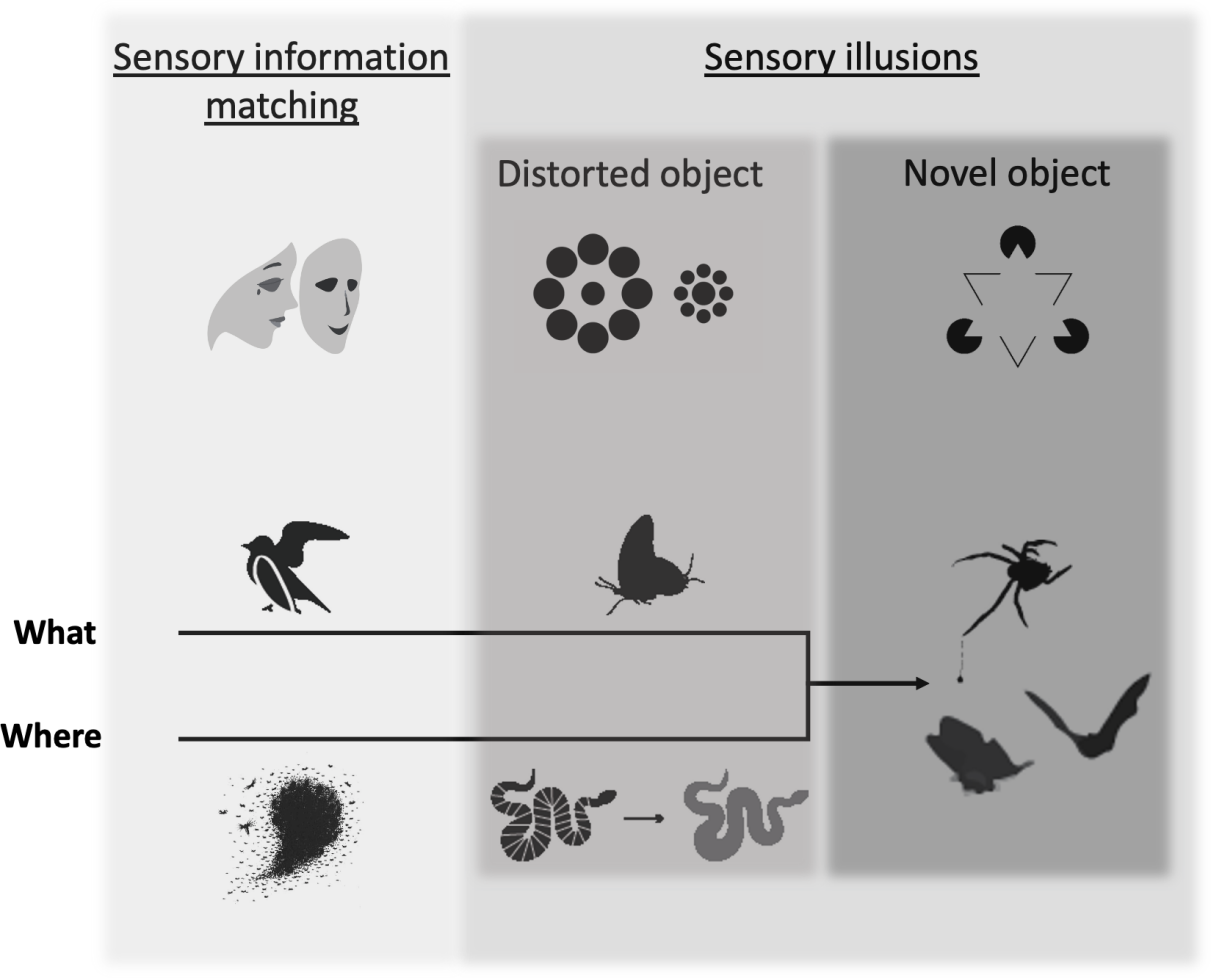
The deception framework. Deceptive traits can be broken into two primary categories: those that faithfully mirror the traits of another object (sensory information matching) and those that create a discrepancy between reality and perception (sensory illusions), which we further break down by how they affect object formation (distorted object, novel object). Shaded boxes indicate the distinction between the two major categories and the two subcategories of sensory illusion. Increasingly dark shades of gray indicate a hypothesized increasing specificity of the deceptive trait to a particular perceiver’s sensing system. The top row of icons illustrates each category according to known deceptive effects for human observers: (1) sensory information matching–an actor wears a mask to appear as someone else; (2) distorted object–Ebbinghaus illusion (the interior circle on the left appears smaller than the interior circle on the right, although they are the same size), (3) novel object–Kanisza triangle illusion (a human observer sees a white triangle, although it is not actually present). Deceptive traits can fool a perceiver along the “where” or “what” axes of object processing, which eventually converge in novel object sensory illusions. Examples from the animal literature include (from left to right): sensory information matching = a bird’s broken wing display misleads a predator about its vulnerability and fish schools prevent a predator from localizing an individual; distorted object = false heads in a butterfly misdirects predator attack and flicker-fusion in a snake makes it more difficult to track in motion; novel object = bolas spiders draw in males with female moth-like pheromone and moth tails create alternative targets for echolocating bats.

The world is full of deceit. Across systems and sensory modalities, animals convey information intentionally or inadvertently (Bradbury & Vehrencamp, 2011). Many interspecific interactions are marked by the transmission of misrepresentative or misleading signals (Edmunds, 1974; Dawkins & Krebs, 1978; Searcy & Nowicki, 2005; Schaefer & Ruxton, 2009; Ruxton & Schaefer, 2011; Carazo & Font, 2014). Deception—the transmission of misleading or distorted information to a perceiver, to the benefit of a deceiver, is perhaps most obvious in predator–prey interactions. Cuttlefish (*Sepia*) rapidly change their coloration and texture in response to predator threat (Hanlon & Messenger, 1988; Langridge, Broom & Osorio, 2007), palatable tiger moths (*Arctiinae*) produce warning clicks that mimic those of their chemically-defended counterparts (Barber & Conner, 2007), and bolas spiders (*Mastophora*) lure in moth prey with female pheromones (Stowe, Tumlinson & Heath, 1987). Examples of deception can be found in nearly every animal sensory system and are common across taxa (Searcy & Nowicki, 2005). Previous reviews on deceptive traits in animals have provided substantial insight into the evolutionary underpinnings of this strategy (Bond & Robinson, 1988; Schaefer & Ruxton, 2009; Carazo & Font, 2014; Caro, 2014; Mokkonen & Lindstedt, 2016; Font, 2019). A review by Kelley & Kelley (2014a) reinvigorated the conversation about deception, bringing many deceptive strategies under the umbrella of illusions and prompting a round of debate in the field (Kelley & Kelley, 2014b; Kemp & White, 2014; Merilaita, 2014; Ryan, 2014; Stevens, 2014; Théry, 2014). This article and resulting comments provided a critical contribution to the discussion. A unified framework that provides a sensory metric by which to distinguish deceptive strategies is still needed, however. Such a framework will help researchers identify the deceptive strategy being employed in their system and the evolutionary pressures that have driven these deceptive traits, as well as the pressures these traits have exerted on the sensory systems of their perceivers (Fig. 1).

Our framework draws from predator–prey interactions, as these are often emblematic of the most high-stakes encounters (Dawkins & Krebs, 1979). Predator–prey battles occur between animals with sometimes differing sensory systems that are entwined through evolutionary conflict (Endler, 1992). Regardless of the sensory system, to navigate their world, predators and prey must divide their surroundings into distinct, cohesive units to extract relevant figures (objects of interest) from background (surrounding scene) (Feldman, 2003). Classic cognitive neuroscience studies have found evidence for “what” (*e.g.*, shape, color) and “where” (*e.g.*, location, motion) pathways that transmit visual stimuli separately before the information is reintegrated in a processing center to create a complete perceived object (Mishkin, Ungerleider & Macko, 1983; Rauschecker, 1998). While the exact function and format of these pathways has since been redefined (for review, see Freud, Plaut & Behrmann (2016)), the general principle that an object’s physical attributes and location in space are critical components of perceptually forming a complete object still stands (Bizley & Cohen, 2013; Kimchi et al., 2016). Moreover, these basic tenets of object formation seem to be conserved across sensory systems and taxa. We primarily focus on vision and audition in this review, due to the greater wealth of studies in these sensory systems. For a discussion of similar object formation processing in vibrissae sensorimotor sensing, see Diamond et al. (2008); for electroreception sensing, see von der Emde & Schwarz (2000) and von der Emde et al. (2010); and for olfaction, see Wilson & Sullivan (2011). The specifics of neurobiology are outside the scope of this article, but we use the commonality of perceptual processing to structure our discussion of deceptive traits. We distinguish traits that affect the processing of what an object is from traits that affect the processing of where an object is. Using these two categories, we create a distinction between traits that mirror the sensory information of another object to mislead and traits that make use of sensory shortcuts and perceptual biases (Schaefer & Ruxton, 2009) to create a discrepancy between physical reality and perception, *i.e.,* sensory illusions (Gregory, 1997; Kelley & Kelley, 2014a). Finally, we suggest that sensory illusions can be divided into those that distort signaler characteristics and those that create the perception of a novel object. We suggest that sensory information matching requires the least specificity to a particular perceiver, as it relies mainly upon high fidelity matching of another object along a given sensory channel. Sensory illusions, on the other hand, are more closely tailored to the sensory system of their particular perceiver, as they rely upon manipulation of the perceiver’s sensory system to produce an effect. Of course, traits are likely to have different effects on diverse perceivers, based on senosry and cognitive processing. We therefore take a perceiver-perspective, using examples from the literature that test specific, biologically-relevant perceiver-deceiver interactions, but do not rule out other roles that deceptive traits may play for other perceivers. Additionally, the natural world defies strict categorization. We here advance this framework not as an immutable set of labels, but as an organizational structure that researchers can use to investigate traits within their study systems, through the lens of object formation.

By considering how a deceptive trait influences object formation (previously introduced by Gregory (1980)), and whether it affects the perceiver’s assessment of where the object is in space or what the object is, researchers can achieve a better understanding of the evolutionary drivers of prey traits and begin to predict how these traits might affect predator–prey dynamics. Some traits thwart object formation processes entirely, preventing segregation from background (see *Object formation* section below) and leading to crypsis. We focus our review to traits that operate after an object is formed, however, as these traits have evolved to manipulate a particular facet of object formation (namely, what or where the object is), rather than erasing the object entirely from the perceiver’s perspective. Traits that prevent object formation, leading to crypsis, are numerous and fascinating and we encourage the reader to look to previous reviews on this topic (for example, see Stevens & Merilaita (2009)). We suggest that the present framework will provide a useful tool to further probe the complex evolutionary dynamics between deceivers and perceivers post-detection. Through this lens, we can gain insight into a potent and pervasive biological interaction that has shaped both animal traits and sensory systems across time.

### Object formation

As animals navigate their world, they must continually parse relevant information from a cacophony of stimuli. Animals segregate object from background by perceptually binding (grouping) elements in a scene that arise from a similar point in space at a similar time (auditory system: Bregman, 1990; auditory & visual system: Kubovy & Van Valkenburg, 2001; visual system: Robertson, 2012; chemosensory system: Stevenson, 2014). This process is generally accomplished following gestalt principles, where elements are grouped based on their proximity, likeness, continuity or closure, and common direction/speed (Wertheimer, 1938; Koffka, 1935; and for auditory objects, see Dent & Bee (2018)). Additionally, while object formation can be a pre-attentive process, attention and memory can also play an important role, regardless of the perceiver’s apparent cognitive abilities Wertheimer, 1938; Koffka, 1935; Kubovy & Van Valkenburg, 2001; Pressnitzer et al., 2008; Kondo et al., 2017; Winsor et al., 2021. Perceptual grouping mechanisms and the associated figure-ground segregation that leads to object formation have been demonstrated across diverse taxa and sensory systems, including birds (Regolin & Vallortigara, 1995; Dent & Bee, 2018; Suzuki, 2020), fish (Fay & Popper, 1998; Engelmann et al., 2008; Salva, Sovrano & Vallortigara, 2014), amphibians (Dent & Bee, 2018), insects (Horridge, Zhang & O’Carroll, 1992; Schul & Sheridan, 2006), and mammals (Hubel & Wiesel, 1962; Simmons et al., 1974). Thus, while different sensory systems and taxa may use varying mechanisms for receiving and integrating incoming information, the propensity for perceptual grouping seems to be highly conserved evolutionarily. Selection on traits that obstruct correct object formation for a generalized perceiver’s sensory system may therefore be strong across different taxa.

## Survey Methodology

This article is intended to review concepts and data from both recent and old sources to arrive at a novel, theoretical contribution to the deception literature. The interdisciplinary nature of this topic is critical to the relevance of the review and to its applicability to a broad range of fields, including animal behavior, psychology, neurobiology and evolutionary biology. We performed extensive literature searches in Google Scholar in the USA between the years 2018–2023 that were unconstrained by animal taxon, date, or peer-reviewed journal. We used search terms including “animal deception”, “deceptive traits”, “dishonest traits”, “sensory illusion”, “animal illusions”, “cognitive illusion”, “auditory illusion”, “visual illusion”, “where/what pathways”, “mimicry”, “animal bluffing”, “unreliable signals”, “predator confusion”, “predator prey evolution”, “object formation”, “animal/insect object formation”, “perceptual grouping”, “auditory scene analysis”, “chemical scene analysis”, and “sensory exploitation”. We also incorporated papers that were cited in articles that we identified as important to the foundation of our work.

## Exploring the Framework

### Using sensory information matching

Traits can mislead a perceiver by closely reproducing the sensory information of another object that, when occurring in other contexts, conveys reliable information. Thus, these deceptive traits do not manipulate sensory processing by the perceiver, but rather they deceive by matching the attributes of another object or animal. Previously, authors have referred to this phenomenon as dishonesty (Dawkins & Guilford, 1991; Searcy & Nowicki, 2005), bluffing (Bond & Robinson, 1988; Caro, 2014), or parasitic signaling (Carazo & Font, 2014). We take no issue with these terms, but aim to build a broader descriptive category to encapsulate the variety of traits that use a similar deceptive approach. We here outline a few examples, representing multiple sensory systems. We begin with strategies that lead to the misidentification of the signaler (what) and proceed to examples that lead to the mislocalization of the signaler (where).

### Deception of “what”

#### Feigning injury or death

**Figure 2:**
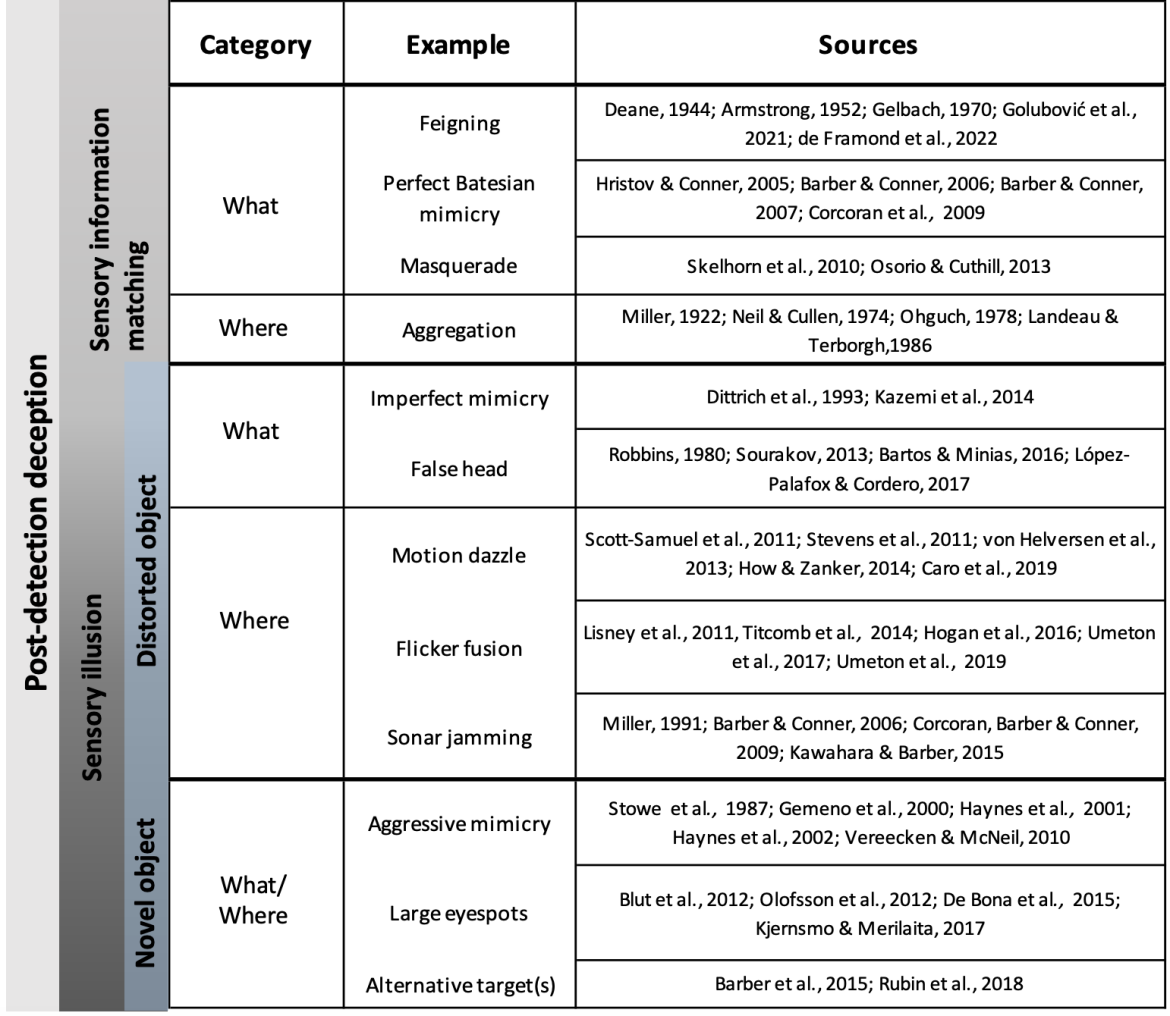
Examples of deceptive traits in animals. Common examples of each deceptive category are listed next to the object formation axis that they thwart (what or where axes). Primary literature sources for each example are listed in the rightmost column and full citations can be found in the References section.

At least 52 bird species (de Framond et al., 2022) are known to perform broken-wing displays to divert approaching predators from their nests (Deane, 1944; Armstrong, 1954; Caro, 2014; de Framond et al., 2022) (Figs. 1, 2). These displays contain all the visual information of an injured bird—wing held askew, bird running instead of flying, *etc*.—but the injury is fictional (*sensu* Gregory (1980)). Similarly, death-feigning behavior to discontinue predator attack is widespread across taxa (Humphreys & Ruxton, 2018). From beetles to birds, many animals have evolved anti-predator traits that closely overlap the behavioral and physiological manifestations of death, often including some form of body limpness or stiffening and even crossing sensory systems to include defecation and blood spitting (Gehlbach, 1970; Humphreys & Ruxton, 2018; Golubović et al., 2021). In these cases, the discrepancy between reality and perception is minimal. That is, the deceiver’s traits are functionally identical to the receiver’s perception of the trait.

#### Masquerade

Many animals have evolved traits that closely resemble an object that the predator would not want to attack, such as twigs and leaves (Fig. 2) (Nieder, 2002; Skelhorn et al., 2010; Osorio & Cuthill, 2013). Here, the animal’s traits closely overlap the attributes of inedible object (rather than another animal) to promote misidentification. This strategy is distinct from crypsis in that the deceptive trick does not rely on environmental background. That is, even if the perceiver detects the signaler (*i.e.,* separates the object from background) (Stevens & Merilaita, 2009; Merilaita, Scott-Samuel & Cuthill, 2017), the signaler is still protected by its masquerade. This effect has predominantly been shown in the visual system, often using domestic chicks (*Gallus gallus*) as model predators. A foundational study found that twig-resembling caterpillars presented on a blank background elicited a slower approach and more cautious handling by chicks with previous experience of twigs than those without (Skelhorn et al., 2010). Crab spiders that (to human observers) resemble bird feces (*Phrynerachnae celonica*) experienced fewer attacks and longer latency to attack by chicks than size and shape-matched models with a different color pattern (Yu et al., 2022a). In a marine system, cephalopods (*Sepia officinalis*) adaptively change the texture and color patterning of their skin to mirror objects in their environment, such as rocks (Panetta, Buresch & Hanlon, 2017). Predators themselves also make use of masquerade: the same crab spider ambushes flower-visiting arthropods that mistake it for a non-threatening object (Yu et al., 2022b) and ghost mantids (*Phylocrania paradoxa*) more successfully captured crickets (*Acheta domesticus*) that had previously experienced dead leaves (and found them to be innocuous), compared with leaf-naïve crickets (Skelhorn, 2018). Masquerade therefore provides a cognitive foil against perceivers, such that they are led to misinterpret the deceiver (Pembury Smith & Ruxton, 2020). Future work exploring masquerade in other sensory systems will provide important additional insight.

#### Perfect Batesian mimicry

Animals also often mimic other animals that a predator would not want to attack, namely, noxious or dangerous prey (Fig. 2) (Poulton, 1980). Batesian mimicry, where a palatable organism closely resembles a toxic one, can manifest as color patterning (Bates, 1862; Ruxton, Sherratt & Speed, 2004; Darst & Cummings, 2006; Leavey et al., 2021), locomotor behavior (Srygley, 1999; Outomuro et al., 2016) and acoustic signals (Hristov & Conner, 2005; Barber & Conner, 2007) that mirror those of their chemically defended models (Fig. 2). Mimics can fall along a wide spectrum of signal overlap accuracy, from imperfect (see *Sensory illusions* section below for a discussion of imperfect mimicry) (Dittrich et al., 1993; Edmunds, 2000; Dalziell & Welbergen, 2016) to something closer to perfect mimicry (precise matching). A tighter overlap in the display between model and mimic seems to confer increased protection (Dittrich et al., 1993; Mappes & Alatalo, 1997). While precise matching can lead to vulnerability if the model’s traits or distribution changes (Joron, 2008), it can also make model and mimic almost indistinguishable, even with longer information gathering and processing by the perceiver. As has been noted, it can be difficult to determine whether an animal is a high-fidelity mimic to a given perceiver, as doing so requires deep understanding of another animal’s sensory system (Cuthill & Bennett, 1993; Edmunds, 2000). Recent technological advancements have allowed researchers better access to information outside of the human perceptual sphere and to more objectively measure signal overlap across species (Caves, Brandley & Johnsen, 2018; Kelly et al., 2021; Barber et al., 2022).

### Deception of “where”

#### Confusing aggregations

One way aggregating animals can mislead perceivers about their location is by sensorially mirroring the conspecifics around them, making it hard for predators to accurately target and capture individual prey, leading to “confusion” (Miller, 1922; Landeau & Terborgh, 1986; Ioannou et al., 2008). Regardless of the sensory system that the perceiver is using, this effect seems to work best when aggregating individuals appear homogenous (Fig. 1) (Goodale, Ruxton & Beauchamp, 2019; Cuthill, Matchette & Scott-Samuel, 2019; for fish schooling examples, see Neill & Cullen (1974) and Landeau & Terborgh (1986); for zooplankton, see Jeschke & Tollrian (2007); for katydid aggregations, see Prakash et al. (2021)). Predators facing groups of visually or acoustically similar animals hesitate more (Schradin, 2000; Prakash et al., 2021) and have lower capture success than predators attacking single or few prey (Ioannou et al., 2008; Brighton et al., 2021) or members of a group that appear different (“oddity effect”) (Ohguchi, 1978; Landeau & Terborgh, 1986). These effects are caused by an increased difficulty in focusing on and tracking one particular prey item amidst alternative similar prey, which could be driven by perceptual processing constraints (Krakauer, 1995; Jeschke & Tollrian, 2007; Goodale, Ruxton & Beauchamp, 2019) or cognitive constraints *via* indecisiveness (Prakash et al., 2021). Some predators have evolved hunting strategies to get around this deceptive grouping behavior. These include seabirds hunting in concert to break up fish schools (Thiebault et al., 2016) or a single raptor diving into a bat swarm and grabbing the individual closest to them (Brighton et al., 2022). Interestingly, none of these strategies seem to allow the perceiver to accurately localize a particular aggregating individual in space, indicating that this may be an exceptionally difficult perceptual task. At this time in the literature, prey aggregation is the only well-supported example of a strategy in the sensory information matching category that leads to deception of “where”. We expect that other examples exist in the natural world, but that they require more directed attention and empirical testing to elucidate.

### Sensory Illusions

Sensory illusions are evoked by traits that take advantage of pre-existing perceptual biases and sensory processing shortcuts in a perceiver’s sensory system to create dissonance with reality (Gregory, 1997; Kelley & Kelley, 2014a; Kemp & White, 2014; Merilaita, 2014; Ryan, 2014; Stevens, 2014; Théry, 2014). In this section, we build upon the seminal work by Kelley & Kelley (2014a; 2014b) and commentors (Kemp & White, 2014; Merilaita, 2014; Ryan, 2014; Stevens, 2014; Théry, 2014) on visual illusions in animals, in addition to basic principles of sensory illusions outlined by Gregory (1997). We make use of the perceptual perspectives discussed in Kelley & Kelley (2014a) and Gregory (1997) to suggest two major types of sensory illusion: traits that distort object characteristics and traits that form entirely novel objects. We believe that many of the sub-categories outlined in Kelley & Kelley (2014a), Kelley & Kelley (2014b) (*i.e.,* illusion of size, illusion of shape, *etc*.) exist within this first category (Gregory, 1997; Kelley & Kelley, 2014a). The second category, novel object, has not to our knowledge been previously described. We suggest that this addition contributes to our understanding of deception, as these traits corrupt both object attributes and spatial information, causing a perceiver to perceptually bind an object that does not truly exist. As with the section above, we aim to highlight examples across sensory systems, although visual illusions are the most commonly studied (Kelley & Kelley, 2014a).

A primary constraint to uncovering sensory illusions has been the limited understanding of mechanisms that underly perceptual processing across organisms. One of the great benefits of studying illusions, however, is the information it can reveal about how the sensory system functions—*via* understanding the conditions under which perceptual flaws emerge (Eagleman, 2001). Research on visual illusions has revealed unexpected overlap between sensory systems comprised of entirely disparate receptive machinery, neurophysiological organization, and evolutionary history. For instance, humans (*Homo*), domestic chicks (*Gallus*), goldfish (*Carassius*), and presently-studied insects all fall prey to the Kanizsa triangle visual illusion, where a triangle becomes apparent between the broken edges of other shapes (Fig. 1) (Kanizsa, 1976; Horridge, Zhang & O’Carroll, 1992; Davis & Driver, 1994; Wyzisk & Neumeyer, 2007; Mascalzoni & Regolin, 2011). We discuss this illusion further in the novel object section below. The study of illusions has also exposed surprising differences in superficially similar systems: while humans, dolphins, and goldfish all demonstrate susceptibility to the Ebbinghaus illusion, where two circles of the same size are surrounded by either larger or smaller circles (Fig. 1), baboons (*Papio*) do not, and dogs (*Canis*), chickens, and pigeons (*Columba*) are fooled in the opposite way from adult humans (Feng et al., 2017; Byosiere et al., 2020). Testing animals with the Ebbinghaus illusion has led researchers to better understand size estimation *via* monocular and binocular processing in humans (Song, Schwarzkopf & Rees, 2011), as well as perceptual processing differences between global-oriented imagers (adult humans and dolphins) (Navon, 1977; Doherty et al., 2010; Murayama et al., 2012) and individual element imagers (pigeons) (Nakamura, Watanabe & Fujita, 2008). Moreover, context-based illusions, such as the Ebbinghaus illusion, seem to be related to predictive processing that diverse taxa use to assess and interpret the world based on prior experiences and expected outcomes (Feng et al., 2017; Leavell & Bernal, 2019). However, after over a century of research, exactly how this illusion works is still not fully understood (Kirsch & Kunde, 2021). We therefore focus here on a few well-described examples of sensory illusions, which we hope will stimulate future work aimed at uncovering previously unrecognized examples along other sensory channels.

### Illusion of “what”—Distorting object characteristics:

#### Imperfect mimicry

Perceivers may make imperfect assessments due to limited physical temporal processing or speed-accuracy tradeoffs, where they make an assumption based on limited information in a noisy environment (Chittka, Skorupski & Raine, 2009). Imperfect mimicry, where traits resemble model organisms but fall short of exact congruence, capitalizes on this constraint (Fig. 2) (Sherratt, 2002). This strategy seems to take advantage of a variety of processing layers, including individual experience, stimulus overshadowing, where salient stimuli are noted in lieu of others to categorize an object (Mackintosh, 1976; Sherratt et al., 2015) and perceptual biases (Schaefer & Ruxton, 2009), where signaler traits evolve to exploit inherent preferences in a perceiver’s sensory system (Ryan, 1990). Hoverflies (Syrphidae) provide a classic example of imperfect mimicry. Many of these mimics are only loosely reminiscent of a dangerous wasp—a model that has reinforced its threat to its predators for the past ∼150 million years (Dittrich et al., 1993; Peters et al., 2017) (Fig. 2). To test the mechanics of imperfect mimicry, Kazemi et al. (2014) conducted a study with blue tits (*Parus caeruleus*) and artificial prey that varied in pattern, shape and color and that were differentially associated with reward. They found that color stimuli were highly salient to these bird predators, while patterning and shape were perceptually ranked lower, possibly due to the unreliability of these cues across distance and angle of observation. While animals might possess innate phobias of certain colors (Rowe & Guilford, 1996) (and therefore inherent prioritization of color cues), this can be overcome (Adamová-Ježová et al., 2016) or reinforced by experience (Skelhorn & Rowe, 2006). Of course, “imperfect” is in the eye of the beholder (Cuthill & Bennett, 1993; Penney et al., 2012). The sensory information conveyed by a deceiver may amount to nearly complete sensory information matching for one perceiver and only partial information matching for another. This again highlights varying effects that a deceptive trait can have for different taxa and sensory systems. In many cases, individualized studies into perceiver-deceiver dynamics may be necessary to clarify the exact type of deception at play.

#### False heads

Animals might also mimic their own body parts to deflect, rather than prevent, predator strikes. Some butterflies possess protruding hindwing structures that they flick while at rest, resembling (to human observers) antennae facing the opposite direction from the butterfly’s real head (Fig. 1, Fig. 2) (Robbins, 1980; Hendrick et al., 2022). Choice experiments using salticid spider predators with Lepidoptera (Sourakov, 2013), and computer-based prey (Bartos & Minias, 2016), indicate that false head structures draw spider predatory attack away from the true head (Sourakov, 2013; Bartos & Minias, 2016). Praying mantises, however, do not seem to be swayed by these alternative appendages (López-Palafox & Cordero, 2017). This disparity in responses highlights the strong effect that perceptual biases and sensory sensitivity can have on the success of a deceptive trait, even within the same modality (*i.e.,* vision). Salticid spiders, with their unusual visual acuity, might make more use of cephalic detail stimuli to identify where to strike their prey (Bartos & Minias, 2016), while the praying mantis’ motion detection-oriented visual system (Nityananda et al., 2018; Nityananda et al., 2019) seems to not be so easily fooled by the false head elements. Importantly, however, when it does work, the deceptive quality of this trait appears to be misleading the predator as to which end to attack –that is, where the true head is. A similar effect may also occur in some sea snakes (*Laticauda*), which have semi-flattened tail ends that they wave in the water as they forage, in a similar motion to their true head (Rasmussen & Elmberg, 2009). In both the butterfly and snake systems, further work with ecologically-relevant predators is needed to elucidate the role of perceptual bias in the efficacy and maintenance of the false head trait (Hendrick et al., 2022).

### Illusion of “where” –Distorting object characteristics

#### Motion dazzle

Perceivers must make informed assessments of the world based on information that they can take in and process in a given period (Gold & Shadlen, 2007; Leavell & Bernal, 2019). In the visual system, multiple kinds of patterns or markings seem to manipulate this limitation to thwart the perceiver in multiple ways. Some animals have alternating bands of contrasting color that create a motion dazzle effect, causing the predator to misjudge the prey’s speed and direction of escape (Fig. 2) (Thayer, 1909; Pough, 1976; Endler, 1978; Umeton, Read & Rowe, 2017; Umeton et al., 2019). This illusion is likely driven by the temporal/spatial summation employed by the perceiver’s visual system (Castet et al., 1993; Warrant, 1999), as well as other external factors such as distance from the prey, light levels, and background environment (Cuthill, Matchette & Scott-Samuel, 2019; Kodandaramaiah et al., 2020). Certain strategies and constraints of many visual systems may be especially implicated in this illusion. Saccadic eye movements used by animals tracking a moving target could evoke perceptual flaws similar to those found in visual illusions such as the wagon wheel effect (an erroneous perception of backward rotating motion (Purves, Paydarfar & Andrews, 1996; Lisi & Cavanagh, 2015). Additionally, limitations of tracking linear patterns through a narrow aperture (eye) can make the direction of the line difficult to judge, as occurs in the barber pole illusion (a misperception of movement along the length of the pole, rather than perpendicular to it (Adelson & Movshon, 1982; Fisher & Zanker, 2001). In short, these errors can result in mistaken assessment about the direction and speed of an object’s movement (Diener et al., 1976; Castet et al., 1993; How & Zanker, 2014; Caro et al., 2019). Studies with human “predators” and virtual striped “prey” provide evidence for motion dazzle, given high enough speeds or rotational movement (Stevens, Yule & Ruxton, 2008; Scott-Samuel et al., 2011; Stevens et al., 2011; von Helversen, Schooler & Czienskowski, 2013; Hogan, Cuthill & Scott-Samuel, 2016). Research has shown that some birds have trouble capturing moving, patterned prey (Hämäläinen et al., 2015) and tanabid flies have greater difficulty landing on horses wearing striped coats than horses wearing solid color coats (Caro et al., 2019). More studies with real predators attempting to strike prey against natural, convoluted backgrounds are needed, particularly given the conflicting evidence of this effect against humans playing computer simulations (Hughes et al., 2021).

#### Flicker-fusion

Flicker-fusion can be another effect of striping and color bands, in this case driven by the critical flicker frequency (CFF) of the perceiver’s visual system. CFF can be defined as the fastest rate a visual stimulus can flicker before the visual system fuses it into a continuous percept (Umeton, Read & Rowe, 2017). In a study with praying mantid (*Sphodromantis lineola*) predators and digital prey, narrow-striped prey were detected less often when they moved quickly compared to background-matching prey moving at a similar speed, or narrow-striped prey that moved slowly (Umeton et al., 2019). Vertebrate predators likely suffer a similar effect. Spectral measurements of snakes (*Lampromeltis*) indicate that their alternating color bands blur in motion to generate a uniform color (for instance, black and white stripes blurring to grey) for predators whose CFF is ∼90 Hz, comparable to that of a chicken (*G. gallus*) (Fig. 1, Fig. 2) (Lisney et al., 2011; Titcomb, Kikuchi & Pfennig, 2014). Most raptors have higher CFFs than chickens, but Harris’s hawks (*Parabuteo unicinctus*), common predators of snakes and other reptiles, have CFFs in the human and other mammal range (∼25–50 Hz) (Jiang, Zhou & He, 2007; Potier et al., 2020). While flicker-fusion can lead to more effective crypsis in motion than background matching prey (Umeton et al., 2019), it could also serve an alternative function of making the prey animal look starkly different in motion than it does when it comes to an abrupt stop and its patterning is plainly seen (Pough, 1976). Studies further investigating the CFF of diverse predators and the survival of striped prey under different motion regimes against different backgrounds will provide more information about the sensory drivers of this anti-predator patterning.

#### Sonar jamming

Temporal integration limitations also function in the auditory system. Some tiger moths and hawkmoths stimulated by bat sonar playbacks (Barber & Conner, 2006; Barber & Kawahara, 2013; Rubin, 2022) and real attacks (Corcoran, Barber & Conner, 2009; Kawahara & Barber, 2015) respond with ultrasonic clicks of their own at such high duty cycles (sound per unit time) that they can jam bat sonar. These high-repetition click streams contain energy across a broad range of frequencies that can disrupt the bat’s auditory system and introduce range estimation errors (Miller, 1991; Barber & Conner, 2006; Corcoran, Barber & Conner, 2009). Bats determine distance by comparing the timing of the outgoing sonar pulse and the returning echo (Simmons, 1973). High duty cycle moth clicks fall within the bat’s integration window and seem to either cause neuronal firing in response to both the moth click and returning bat echo, or suppress neuronal response entirely (Tougaard, Casseday & Covey, 1998; Corcoran et al., 2011). In doing so, these high duty cycle moth clicks can corrupt bat sensory processing, causing this predator to activate its entire capture sequence in the wrong location and miss the moth target (Corcoran et al., 2011; Kawahara & Barber, 2015).

### Novel object formation

Perhaps the most extreme version of manipulating object formation processes is to elicit perception of a whole object that is not truly there. Here, the illusion occurs as an integrated deception across the “where” and “what” axes. Novel object formation is often driven by a signaler’s trait that exists in the location of the perceived object but makes use of perceptual biases and shortcuts in the perceiver’s sensory system to create a complete object of interest fundamentally different from the trait/animal itself. That is, while it may employ similar sensory tactics to mimicry, perfect (sensory information matching) or imperfect (distorted object), it relies upon manipulation of both the where *and* what axes not to corrupt object formation, but to elicit the formation of a complete, phantasmal object. To generate this effect, the deceiver’s traits elicit what Gregory termed “object-hypotheses”. That is, when faced with ambiguous object information, the perceiver extrapolates from the data it has to create a sensical, coherent object (Gregory, 1980). In the Kanisza triangle illusion (Fig. 1), for example, visual perceivers predict the edges of the white triangle to make sense of gaps in the surrounding shapes (*i.e.,* the triangle is overlaying the other shapes) (Gregory, 1980; Spillmann & Dresp, 1995). Predictive processing, where the perceiver uses innate or learned expectations of stimuli to more effectively order the world, may be an important component of this type of illusion (de Lange, Heilbron & Kok, 2018; Leavell & Bernal, 2019). We suggest, therefore that novel object formation is effected by deceiver traits that capitalize on the perceiver’s perceptual grouping processes and object-hypotheses to form an object that *is not there*. Thus, this category of deception likely creates the widest gap between reality and perception. Novel object illusions may therefore provide some of the most detailed insight into the nuances of a perceiver’s sensory system, perceptual biases and natural selection influences.

#### Novel object aggressive mimicry

Some predators create novel objects by making use of aggressive mimicry to lay a sensory trap (Christy, 1995; for reviews, see Jackson & Cross (2013) and Pembury Smith & Ruxton (2020)). Bolas spiders release volatile compounds that comprise many of the same chemicals in similar blend ratios to the sex pheromones of female bristly cutworm moths (*Lacinipolia renigera*) (Fig. 1, Fig. 2) (Gemeno, Yeargan & Haynes, 2000). As the spider lures in male cutworm moths with its chemosensory trick, it traps any that get too close to its sticky silk bolas (Haynes, Yeargan & Gemeno, 2001). Additionally, these spiders change the relative ratio of their chemical emissions over the course of the night, such that they can catch males of different moth species (Haynes et al., 2002). There is convincing support that male moths perceive a conspecific female from the spider’s emissions, as ∼90% of all moths captured by these spiders are males of their select target species (Vereecken & McNeil, 2010). To ensure the placement of this example, experiments will need to be done with these moths to determine whether they are forming perceptual objects, rather than simply responding to a chemical sensory stimulus. One way to test this is to determine whether the moth is using an object-hypothesis by discerning whether it is expecting to find a whole female form at the end of the pheromone plume. Research on codling moths (*Laspeyresia pomonella*) has shown some evidence of this expected-female effect in that males preferentially orient towards, and interact with, pheromone cues that are paired with a visual stimulus of a female moth (Castrovillo & Cardé, 1980). Behavioral studies in the bolas spider-cutworm moth system indicate that male moths orient to the spider that is releasing the chemical signal, rather than the bolas (Eberhard, 1977). More studies into the male’s perceptual expectations would elucidate whether the chemical emissions from the spider are effectively generating a phantom female for the deceived moth.

#### Large eyespots with glint

Rather than drawing prey in, some animals use novel object illusions to deter predators. Along the visual axis, large eyespots possessing ultraviolet reflective “sparkle” highlights can delay or prevent predatory attack, presumably because they incite perception of an intimidating observer. That is, these eyespots seem to function by using object-hypotheses to build the threat of an entire predator based on the perception of large, realistic eyes. In a bird-prey study, Blut et al. (2012) found that lepidopteran models with large eyespots possessing sparkle provide an approximately 20% increase in survival benefit, *versus* models with eyespots of the same size but lacking the sparkle. Moreover, eyespots with sparkle in an anatomically correct location (approximating reflective glints in three-dimensional eyes) *versus* the same sparkle in biologically unnatural locations lead to increased survival benefit. In another study, birds presented with displaying peacock butterflies (*Inachis io*) produced alarm calls and demonstrated reluctance to recommence foraging after this encounter (Olofsson et al., 2012). Realistic eyespots also appear to work underwater. Kjernsmo & Merilaita (2017) found that threespine stickleback fish (*Gasterosteus aculeatus*) were more hesitant to attack prey with eye-like markings than those with non-eye-like, but equally color-contrasted markings, especially when the stickleback had been exposed to predator cues. To create a direct comparison with the putative predator template, De Bona et al. (2015) displayed images of an owl with and without eyes and an owl butterfly with and without its eyespots to foraging passerine birds. Owls with eyes and butterflies with eyespots elicited similar aversive responses, while the models without eyes/eyespots did not produce the same effect. The intimidating quality of large, realistic eye spots seems to be increased by a sudden exposure of these features in a deimatic display (Vallin, Jakobsson & Wiklund, 2007): a surprising reveal of a conspicuous trait that reduces attack (Umbers & Mappes, 2016; Drinkwater et al., 2022). In sum, a growing consensus of literature points to a phantom predator illusion, driven by large, realistic eyespots. These markings seem to function differently from other circular patterns, such as small marginal eyespots (Prudic et al., 2015), or contrasting concentric circles that function to redirect attack (Stevens, 2005; Kjernsmo, Grönholm & Merilaita, 2019). More research will need to be done to determine whether the deflection effect of marginal eyespots are generated by object corruption or the formation of a non-vertebrate predator phantom object.

## An Example of the Framework’s Utility

We posit that our framework will be a helpful tool for researchers to evaluate how a trait is affecting object formation processes in their system, interrogate the mechanism underlying this effect, and subsequently predict traits in other taxa that have evolved to generate a similar deceptive impact. Here, we use the bat-moth system as an example. Some saturniid moths have long hindwing tails that end in twisted and cupped tips. Prior to experimental study, it was unknown what role these tails served (Janzen, 1984). Mating and predator–prey experiments have revealed that these tails are not used for sexual selection (Rubin & Kawahara, 2023), but rather they decrease successful capture by bats by deflecting bat attack to these non-essential appendages (Barber et al., 2015; Rubin et al., 2018).

How do long tails with twisted and cupped ends redirect bats? Tail ends rotate behind the moth as it flies and seem to manipulate the echolocation sensing system by reflecting bat sonar to create their own appreciable echo(es) (Barber et al., 2015). As a result of this structure, tails deflect bat attack, with increasingly long tails increasingly drawing predatory strikes to this posterior region, allowing the moth to get away (Rubin et al., 2018).

Which deceptive effect might be at play here? We begin at the sensory illusion junction in the framework, as these tails are creating a discrepancy between reality and perception. That is, they are taking advantage of sonar processing to mislead bat predators, rather than sensorially matching another object. *Distorted object:* tails could be elevating escape success of the moth by creating an enlarged echoic cloud (Janzen, 1984; Lee & Moss, 2016; Rubin et al., 2018). *Novel object:* tails could be creating their own appreciable echoic targets, separate from the echoes generated by the rest of the wings (Rubin et al., 2018). In the case of distorted object, echoes reflected off the moth’s body, wings, and tails could integrate to form the perception of an enlarged object (deception along the “what” axis) (Lee & Moss, 2016; Rubin et al., 2018). If this were the case, it might be expected that the bat would target the center of this echoic cloud to have the best chance of striking the moth’s body and suppressing its prey. Behavioral experiments show that bats only attack this region (just behind the abdomen) ∼25% of the time, however, while they direct ∼75% of their attacks either towards the abdomen/forewings or the tail ends, indicating the perception of distinct alternative targets (Rubin et al., 2018) and thus a novel object illusion (Fig. 1).

How does parsing these different types of sensory illusion improve our understanding of the evolutionary dynamic between these predators and prey? Differentiating these deceptive effects is critical for understanding the underlying mechanism of this anti-predator strategy and the selective pressures that have shaped it. In this system, behavioral analysis using live bats and 3D sonar beam reconstruction techniques could aid in differentiating object distortion from novel object formation. Here, the sonar beam provides a window into object processing and decision-making by the bat, as the placement and direction of the bat’s acoustic gaze can indicate where it is focusing (Surlykke, Ghose & Moss, 2009; Simmons, 2014). Additionally, the bat’s predatory behaviors (*i.e.,* where it directs its attack) can be used to interpret whether it perceives multiple prey targets or one.

Perhaps most importantly, distinguishing distorted object and novel object illusions might allow us to better understand extant traits and predict traits that other nocturnal arthropods have evolved to thwart echolocating predators. For instance, if the illusory effect of tails is a distorted perception of size, this would indicate the biological importance of limited image resolution by bat sonar sensing (Geberl, Kugler & Wiegrebe, 2019). This could lead to the hypothesis that nocturnal insects extending components of their body in flight, for example, beetle elytra, might be evolutionarily maintained by conveying a general acoustic image of a larger prey. Alternatively, if the illusory effect is novel object formation, this would lead to the prediction that traits in other animals have evolved to act as echoically reflective lures, such as katydids extending their hindlegs and closing their hindwings when evading bats (Kilmer et al., 2010).

## Conclusions

Investigating deceptive traits through a lens of object formation –and particularly breaking object formation down into its principal “what/where” components –can illuminate the selective role that perception has played in driving signaler traits across taxa, and vice versa (Dawkins & Guilford, 1991). As Jakob von Uexküll, a founding mind of the sensory ecology field, so eloquently put it: “If the moth were not batlike its life would soon be over” ((Von Uexküll, 2010) [1934], p. 207). Predators and prey across the Tree of Life have become intimately intertwined through evolutionary conflict and often find their processing of the world vulnerable to manipulation by one another, possibly even escalating to an arms race (Dawkins & Krebs, 1978). Current evidence from the literature (outlined in the examples we have provided in this manuscript) seems to indicate that deceptive traits are increasingly tailored to their particular predators’ sensory systems, leading to increased specificity and deceptive efficacy across the framework’s structure (Fig. 1). Deceptive traits may therefore function as engines of diversification. More studies investigating deceiver/perceiver evolution through the lens of object formation processes would provide important tests of this hypothesis. We encourage researchers to pursue these tests across a wide range of taxa (invertebrate to vertebrate) and ecological systems (terrestrial to marine), using naturalistic predator–prey interactions. Understanding the sensory and cognitive mechanisms that animals use to order their world and by which they can be fooled will help elucidate the evolutionary drivers of traits of interest and allow us to predict their future trajectory, as well as uncovering deceptive traits yet unknown. Such studies thus hold the power to reveal previously unexplored evolutionary dynamics between deceivers/perceivers and to further probe sensory systems, familiar and foreign to our own.

## Additional Information and Declarations

### Data Availability

The following information was supplied regarding data availability: This is a literature review.
